# Temporal coherence structure rapidly shapes neuronal interactions

**DOI:** 10.1038/ncomms13900

**Published:** 2017-01-05

**Authors:** Kai Lu, Yanbo Xu, Pingbo Yin, Andrew J. Oxenham, Jonathan B. Fritz, Shihab A. Shamma

**Affiliations:** 1 Department of Electrical and Computer Engineering, Institute for Systems Research, University of Maryland, College Park, Maryland 20742, USA; 2 Department of Psychology, University of Minnesota, Minneapolis, Minnesota 55455, USA; 3 Department of Cognitive Studies, École Normale Supérieure, 75005 Paris, France

## Abstract

Perception of segregated sources is essential in navigating cluttered acoustic environments. A basic mechanism to implement this process is the temporal coherence principle. It postulates that a signal is perceived as emitted from a single source only when all of its features are temporally modulated coherently, causing them to bind perceptually. Here we report on neural correlates of this process as rapidly reshaped interactions in primary auditory cortex, measured in three different ways: as changes in response rates, as adaptations of spectrotemporal receptive fields following stimulation by temporally coherent and incoherent tone sequences, and as changes in spiking correlations during the tone sequences. Responses, sensitivity and presumed connectivity were rapidly enhanced by synchronous stimuli, and suppressed by alternating (asynchronous) sounds, but only when the animals engaged in task performance and were attentive to the stimuli. Temporal coherence and attention are therefore both important factors in auditory scene analysis.

## Abstract (web summary)

One can easily identify if multiple sounds are originating from a single source yet the neural mechanisms underlying this process are unknown. Here the authors show that temporally coherent sounds elicit changes in receptive field dynamics of auditory cortical neurons in ferrets only when paying attention.

The ability to recognize a target source and segregate it from a complex mixture of sounds is a requisite skill for sensory perception in realistic cluttered environments. The ease with which humans and other animals perform this feat is remarkable. A series of recent studies have suggested that two factors play key roles in this process: the temporal coherence principle and attention. Temporal coherence refers to the postulate that all acoustic features of a sound generated by a single source fluctuate coherently in power over time, and that an attentive listener exploits this coherence to bind and segregate these features from a mixture that contains other temporally incoherent features produced by independent sources in the environment^1^.

Evidence for this role of temporal coherence has been demonstrated in psychoacoustic, brain imaging and computational studies, but not in single-unit neurophysiological studies. For example, psychoacoustic experiments have demonstrated that sequences of synchronous tones were not perceptually segregated, even with large frequency separations^2^ or when one of the sequences is stationary while the other fluctuated slightly in frequencies^3^. Temporal coherence also explained why a few synchronous tone sequences perceptually pop-out even in the midst of a dense background of random tones^4^, and why prominent electroencephalogram responses to these synchronous tones emerge even in the absence of other distinguishing features such as global changes in signal power or local tone densities^5^,^6^. Finally, temporal coherence has also been demonstrated to play a role in co-modulation masking release^7^,^8^ and its dynamics have recently been imaged in the primary auditory cortex^9^.

There are three key ingredients to explain how temporal coherence and attention could be implemented and exploited to disentangle complex sound mixtures such as speech and music: (1) Coincidence operations between pairs of neuronal responses encoding various acoustic features (for example, frequencies, pitches or locations), computations that have been widely invoked for many decades^10^,^11^,^12^,^13^. (2) Binding of coincident responses into one group representing a perceptual source, while segregating it from other non-coincident (or incoherent) responses. One conception of these associative processes is illustrated by the schematic of Fig. 1a. It postulates that neurons with highly correlated responses form cooperative (excitatory) connectivity that can mutually enhance their responses. By contrast, highly uncorrelated (or temporally incoherent) activity leads to competitive (inhibitory) connectivity that mutually suppresses the overall responses while emphasizing the differences between them^14^,^15^. Finally, (3) adaptive connectivity is postulated to require attentive listening to materialize, a conjecture based on previous findings that cortical responses and tuning properties remain largely unchanged during passive listening^16^,^17^.

The experiments described here sought neural evidence of temporal coherence by measuring interactions among neurons, which may indirectly reflect connectivity changes, such as the modulation of response rates and spiking correlations among simultaneously recorded neurons, while stimulating them for several seconds with synchronous (SYN) or alternating (ALT) tone sequences as illustrated in Fig. 1. The spectrotemporal receptive fields were also estimated immediately following the tone sequences by stimulating with a random cloud of tone pips (Fig. 1b) to assess further the evidence for facilitative or suppressive interactions induced by the preceding tone contexts. All measurements were conducted while animals were in passive and in behaviourally attentive states to assess the role of attention in facilitating the adaptive effects. The behavioural tasks were specifically designed to have the animals attend globally (equally) to all sound components, hence allowing the temporal coherence structure of the sequences to influence the relative strength of the responses. The results we report here demonstrate that attentive listening to temporally coherent or incoherent sounds induces rapidly modulated effective interaction among neurons that can be measured by a change of receptive fields, response rates, and of spiking correlations among simultaneously recorded neurons.

## Results

### Design of the behavioural task and acoustic stimuli

Responses were recorded from the primary auditory cortex (A1) of three ferrets. Two ferrets (146 and 116 cells recorded in each) provided the bulk of the data describing the adaptive phenomena in this section. The third ferret (39 cells) served to provide additional data as controls for stimuli that varied in tone rates and overlap.

**Figure 1:**
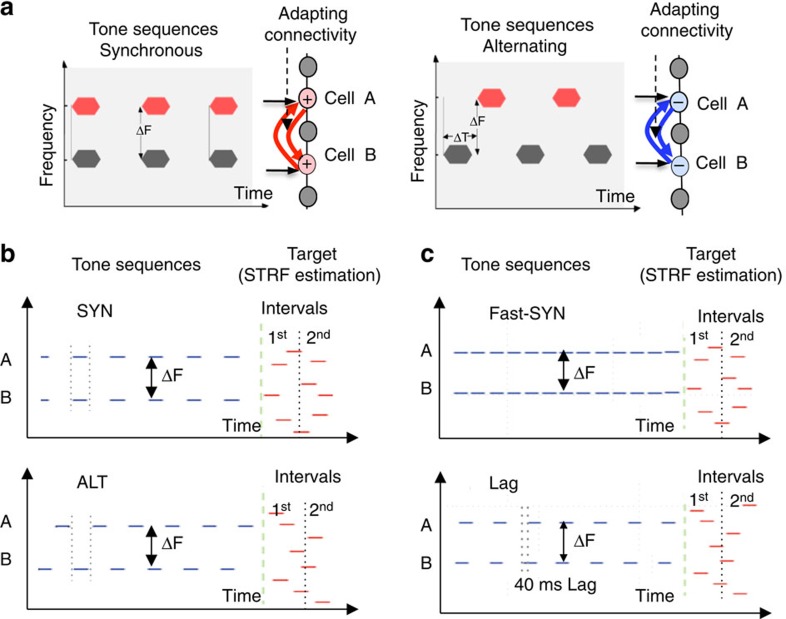
Experiment design and the temporal coherence principle. (**a**) Rationale for the design of the experiments. Synchronous and alternating tones are perceived differently even when the tones are well separated in frequency: the former are perceived as one source, while the latter are perceptually segregated into separate streams. It is hypothesized that the two types of tone sequences induce differential rapid changes in connectivity among the neurons they drive. We conjecture that when coherently driven by synchronous tones, neurons (cell A and cell B) tuned to the two frequencies rapidly form mutually excitatory (cooperative) connections (red arrows). However, when they are driven by alternating tones, we predict that neurons rapidly form mutually suppressive (competitive) connectivity (blue arrows). It is further hypothesized that engagement of attention is necessary to induce these changes in functional connectivity. (**b**) Experimental stimuli. A trial consisted of synchronous (SYN) or alternating (ALT) sequences of 100 ms tones that were followed by a cloud of random tone pips. The frequency of the B tone was fixed at or near most of the CFs of simultaneously recorded neurons in a given experimental neurophysiological recording session. The frequency of A tones changed randomly from one trial to another to be 24, 12, 6 or 3 semitones above or below the B tone. Animals were required to withhold licking during tone sequences and to respond by licking the waterspout following the onset of the random tones (Target). Responses to these random tones in the target tone cloud were used to measure the STRFs of the recorded neurons. (**c**) Experimental stimuli for fast-SYN and Lag. Stimuli for two control conditions: Fast-SYN consisted of the same stimuli as SYN, except that the rate of tone presentation was doubled to match the rate of the tones in the ALT condition. In the Lag stimulus, there was a 40ms time-lag between the tones in the A and B streams.

Ferrets were trained to listen attentively to two-tone sequences (context) without licking the waterspout until the onset of the target tone-cloud stimulus, as depicted in Fig. 1b. They were then rewarded for licking within a short time-window during the target, thus providing an assessment of the overall attentive state of the ferrets to the context tones. Each recording session consisted of three blocks: quiescently listening, followed by active task performance (see Methods section for details), ending with another quiescent listening block immediately following the task.

The ALT and SYN tone sequences can be perceived as segregated or integrated depending on the tone separation (ΔF in Fig. 1a) and other factors which have been studied previously in humans^18^ and in ferrets^19^. Therefore, the ferrets may have perceptually experienced the sounds much like humans do, that is, as being segregated in the ALT context for large ΔF separations (Fig. 1a) and integrated during the SYN context^2^. The design of the behavioural task here, however, did not seek to direct the ferrets' attention to one sequence or another, but rather to have them attend globally to all the context and target sounds. If the ferrets had attended selectively to one of the tone sequences (for example, in the ALT context), it might have resulted in an enhancement of the responses due simply to the attentional focus^16^ and not necessarily to any streaming processes, thus confounding the interpretation of the results.

Figure 1b illustrates the structure of the stimuli in a trial. The ‘context' portion consisted of sequences of two tones (‘A' and ‘B') that were randomly selected on different trials to be either SYN or ALT. The overall duration of the sequences varied randomly between 1.6–3.2 s, and ferrets were required to withhold licking a spout until the regular two-tone sequences changed to a random cloud of tone pips. Licking during the target resulted in a water reward. In each recording session, the frequency of one tone (B) in the sequence was selected to be central, near the characteristic frequency of the majority of simultaneously recorded neurons and remained fixed from trial to trial. In addition to the fixed frequency tone (B), we also presented simultaneously another tone (A) with frequencies that varied randomly from trial to trial over a range of spectral distances (ΔF): 24, 12, 6, 3 semitones above or below the center frequency (B). The target stimulus consisted of random tone pips with a frequency range ±24 semitones around the center frequency (B) that lasted for 1.6 s at the end of the tone sequences in each trial (Fig. 1b). Single-unit responses in primary auditory cortex were recorded in three conditions: initially during passive listening (when no water was present and the animals did not perform the task), followed by the identical stimuli during the behavioural task, and ending with another (post) passive epoch. The responses to the random tone cloud target stimulus were used to measure the spectrotemporal receptive field (STRF) of the neurons immediately following the end of the sequences (see Methods section).

### Context influences neuronal STRFs during task engagement

To assess how the context adapted the responsiveness of the neurons, we computed the STRFs of all units immediately following the presentation of the tone sequences (Fig. 1b), and compared those measured in the first passive session versus the active behaving state. The STRFs were computed separately from the responses to the first and second half of the target random tone pips (first interval 0–800 ms; and second interval 801–1,600 ms; Fig. 1b). Unless specifically stated, all STRFs shown are from the 1st interval of the target. The rationale behind these measurements was that the target stimuli were identical across all task conditions, and hence we reasoned that any differences in the STRFs must have reflected the context (SYN versus ALT preceding sequences) and the behavioural state of the animal (passive versus attentive listening).

The two ferrets participating in these experiments exhibited consistently good performance during the recordings, with the mean percentage of correct trials at 74% (76%±1.4% from 36 recordings for one animal, and 71%±1.7% s.e. from 28 recordings for the other). Analysis of correct trials from all 64 recordings found no significant difference in the behavioural performance of the animals following the ALT trials or SYN sequences (repeated-measures *t*-test: *P*>0.239).

**Figure 2:**
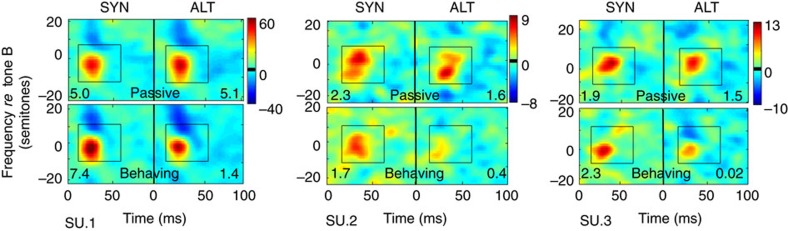
Examples of STRF changes in single units. For each of the three cells, the STRFs in the top panels were measured during the Target epoch after the animal listened passively to the SYN or ALT sequences. The bottom panels were STRFs measured with the same stimuli but during task performance. In these 3 neurons, small differences were seen between the passive STRFs. Significant changes occurred during behaviour with reduction typically following ALT sequences and enhancement following SYN sequences. The average of the STRF within the central highlighted region is indicated in the corner of each STRF.

**Figure 3:**
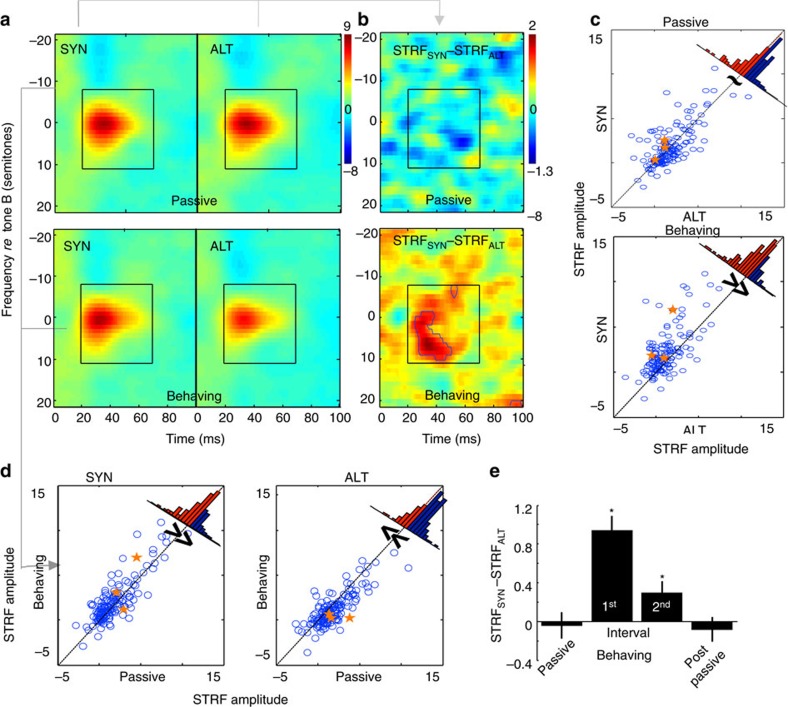
Average STRF changes across all cells. (**a**) Average STRF from all neurons in the passive and behaving states, and in two contexts: preceded by SYN or ALT sequences. The centers of the STRFs from all neurons were aligned at the CF of each neuron, which was typically near the frequency of the B tone sequence. The upper panels display the STRFs in the passive condition. The lower panels show the STRFs during behaviour. The highlighted central region is the window used for quantification and comparison. The same colour-scale is used for all STRFs. (**b**) Average differences in STRFs between SYN and ALT (STRF_SYN_-STRF_ALT_). The upper panel shows that there were no significant STRF differences in the passive condition. During behaviour (lower panel), the STRF differences exhibit significant enhancement. The contours indicate the regions with statistically significant differences from zero (see Methods). The colour scale in these panels is magnified by 4.6 times compared to that of the in **a**. (**c**) Scatter plot of STRF amplitude in SYN versus ALT contexts for each neuron. Each circle represents one neuron. The value on y-axis indicates the mean of the normalized STRF amplitudes indicated by the colour-bar (in the highlighted box of **a**) obtained from SYN conditions, while the value on x-axis indicates the same mean STRF amplitude obtained from ALT condition. The distribution of the points is roughly symmetric during the passive state (top panel). It is biased above the midline in the behaving state (bottom panel). The distribution of all points is shown in the top-right hand corner, with red (blue) denoting the distribution above (below) the midline. Orange stars are the data points computed for the 3 single-units in Fig. 2. (**d**) Scatter plot of STRF amplitude in Behaving versus Passive conditions, measured exactly as in **c** above. For the SYN context, the distribution is biased significantly above the midline. For the ALT context, the distribution is biased significantly below the midline. Orange stars are the data points computed for the 3 single-units in Fig. 2. (**e**) Average STRF changes in SYN and ALT contexts (STRF_SYN_-STRF_ALT_), measured during the first and second 800 ms intervals of the Target tone pips in behaving and passive conditions (pre- and the post-task). STRF differences were larger in the first interval than in the second, but both were significantly larger than zero (indicated by stars, *P*<0.001 for the first interval, *P*<0.01 for the second interval). No significant changes occurred during the pre-task and post-task passive conditions.

Figure 2 illustrates the measured STRFs of three single units in the four different contexts: following ALT/SYN sequences in passive/behaving states. During the passive state (upper row), there were no systematic differences between STRFs following SYN or ALT sequences. The biggest differences appeared instead during behaviour, when the STRFs following the ALT sequences became weaker (suppressed) relative to the passive condition (middle and right cells). Following SYN sequences, the STRFs were either slightly enhanced (left cell) or remained unchanged relative to the passive state (middle and right cells). The changes in these three units are placed in the context of results from all recorded cells later in Fig. 3.

To compute the overall change across many neurons, we selected cells whose characteristic frequencies (CFs) were ±6 semitones around the fixed B-tone sequences (*n*=151). The averaged STRFs from all these cells are shown in Fig. 3a (other neurons were used for analysis of spike correlations as described later). To quantify the changes observed in the STRFs, we computed the STRF peak amplitudes, defined as the averaged response value between 20 and 70 ms (following stimulus onset) and 20 semitones around the CF of the STRF of each selected cell (indicated by the square regions in Fig. 3a). The STRF amplitudes from all 151 neurons were pooled and analysed by two-way repeated-measures analysis of variance (ANOVA), using SYN/ALT as an independent factor and passive/behaviour as a repeated measure. We found a significant effect of behaviour (*F*(1,300)=8.53; *P*<0.004, *n*=151) and a significant interaction between passive/behaviour and SYN/ALT (*F*(1,300)=45.41; *P*<0.001, *n*=151). In the passive state, there were small non-significant differences between the STRF amplitudes following the SYN and ALT contexts (STRF_SYN_ and STRF_ALT_ in top panels of Fig. 3a,b; paired *t*-test: *P*<0.747, *n*=151). However, the STRF peak amplitudes in ALT and SYN diverged significantly when the animal was engaged in the behavioural task (bottom panels of Fig. 3a,b). Specifically, the difference between the STRFs in the two contexts (STRF_SYN_–STRF_ALT_) increased substantially during the behaviour (Fig. 3b; paired *t*-test: *P*<0.001, *n*=151; Bonferroni correction for four comparisons: 0.05/4=0.0125).

These patterns of change are confirmed by the distributions in the scatter plots and histograms of Fig. 3c,d, which summarize the STRF peak amplitude of each cell and from the population as a whole. Thus, during the passive state (top panels of Fig. 3a–c), there were no systematic STRF changes between the two stimulus contexts, and this is evident by the symmetric scatter about the midline in Fig. 3c. By contrast, the effects of SYN versus the ALT contexts were distinctive during the behavioural state (bottom panels of Fig. 3a–c) where the distribution shifted significantly above the midline in Fig. 3c (paired *t*-test: *P*<0.001 with 70% (105/151)).

Figure 3d summarizes the complementary view of the changes, that is, within each context, but across behavioural states. For the SYN contexts (left panels Fig. 3a,d), the majority of points shift above the midline (paired *t*-test: *P*<0.01 with 59% (89/151)), whereas for the ALT context (right panel of Fig. 3a,d), most of the points lie below the midline (paired *t*-test: *P*<0.001 with 77% (116/151)). Finally, note that the three units shown earlier in Fig. 2 are now highlighted by solid orange stars in Fig. 3c,d.

STRFs described thus far were measured during the first time interval (0-800 ms) immediately following the end of the SYN and ALT sequences. Figure 3e depicts the total STRF changes following SYN and ALT contexts during (1) passive state, (2) during first and second 0.8 s intervals of the target tone cloud and (3) during post-task passive state. It is evident that the STRF differences faded within 0.8 s of the target interval, though remaining marginally significant (paired *t*-test: *P*<0.010, *n*=151); they completely disappeared during the second passive session (paired *t*-test: *P*<0.520, *n*=151).

In summary, driving cortical cells with synchronized tone sequences enhanced their responsiveness. The opposite occurred with alternating tones, a pattern that is consistent with the ‘temporal coherence' hypothesis explained in Fig. 1a. None of these changes occurred during passive stimulation, suggesting that task engagement rapidly gates the modulation of these responses as previously reported^17^.

### Contextual effects persist with increasing spectral separation

**Figure 4:**
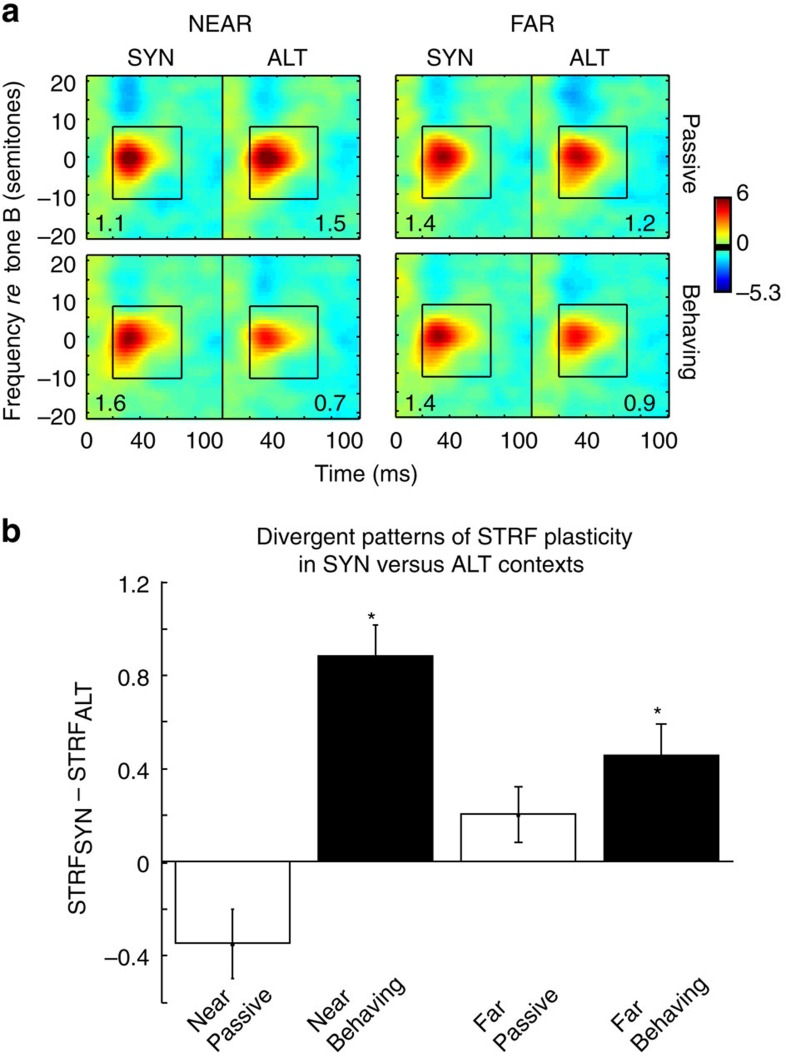
Dependence of STRF changes on sequence spectral separation across all cells. (**a**) Changes in averaged STRFs in SYN and ALT contexts obtained from units with small (NEAR) and large (FAR) spectral separation (ΔF) (left and right pairs of panels, respectively), both in the passive (top) and behaving (bottom) conditions. The same colour-scale is used for all STRFs. As before, STRFs following SYN and ALT contexts exhibited divergent patterns of plasticity in the behaving relative to passive states. (**b**) The average difference of STRF peak amplitudes following SYN versus ALT contexts (STRF_SYN_-STRF_ALT_) for NEAR and FAR conditions, each for passive and behaving conditions. Bars indicate that STRF changes between SYN versus ALT contexts (STRF_SYN_-STRF_ALT_) are significant for both NEAR and FAR conditions (indicated by stars, *P*<0.01 for both), but only during behaviour. Error bars indicate standard error.

We next examined how spectral distance between the two-tone sequences (ΔF in Fig. 1a) might have affected the STRF changes. For this analysis, we segregated and designated the SYN and ALT trials as NEAR (ΔF=±6 and ±3 semitones) and FAR (ΔF=±24 and ±12 semitones). Then the data were analysed with a three-way repeated-measures ANOVA that used NEAR/FAR as one independent variable, SYN/ALT as the second independent variable, and passive/behaviour as a repeated measure. We found a significant interaction between SYN/ALT and passive/behaviour (*F*(1,600)=50.5, *P*<0.001, *n*=151). However, we also found a significant interaction among NEAR/FAR, SYN/ALT and passive/behaviour (*F*(1,600)=21.8, *P*<0.001, *n*=151). Specifically, in the NEAR group (Fig. 4a; left panels), the results were as described earlier (Fig. 3b). A two-way repeated-measures ANOVA showed a significant interaction between SYN/ALT and passive/behaviour (*F*(1,300)=64.0, *P*<0.001, *n*=151). Both NEAR and FAR changes were significant only during behaviour (paired *t*-test: *P*<0.001, *n*=151), although weaker in the FAR conditions (Fig. 4a; right panels). While the interaction between passive/behaviour and SYN/ALT in ANOVA was not significant (*F*(1,300)=3.2, *P*<0.073, *n*=151), there were nevertheless marginally significant effects of behaviour (*F*(1,300)=5.0, *P*<0.025, *n*=151), with STRFs becoming reduced after ALT tones (paired *t*-test: *P*<0.002, *n*=151), and significantly different from those in the SYN context (paired *t*-test: *P*<0.001, *n*=151).

In summary, the changes in neuronal interactions reflecting the SYN and ALT contexts gradually decreased as the tones became more separated in frequency. However, the opposite effects of SYN and ALT sequences persisted even when the tones were separated by over an octave. As before, these effects were absent when the animals listened to the tones passively.

### The effects of tone-sequence rates and time-lag on STRF changes

**Figure 5:**
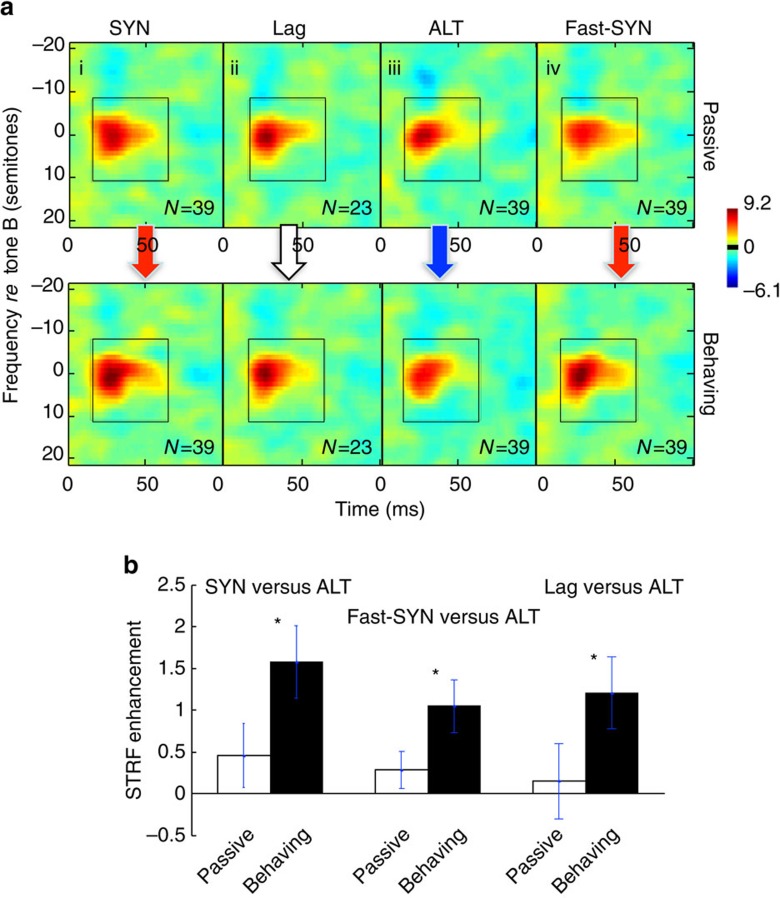
Effects of tone-sequence rates and time-lag averaged across cells. (**a**) Average STRF changes measured simultaneously in four different contexts. (i,iii) SYN and ALT are the usual contexts as depicted in Fig. 1; they exhibit the patterns of STRF enhancement in SYN during behaviour relative to the passive state (red arrow) and suppression in ALT (blue arrow); (ii) The time-Lag condition, where A tone sequences were lagged relative to B by 50% (40 ms). The coherence between the tones was therefore midway between that of SYN and ALT sequences, and the STRFs consequently exhibited little change (white arrow), a pattern similarly midway between the enhancement of the SYN and suppression of the ALT contexts; (iv) A Fast-SYN context in which the tone sequences repeated at twice the rate of the usual SYN condition. The STRF enhancement remained the same as in the SYN condition (red arrow). The two context tones were always separated by 12 semitones, and hence their responses were segregated. (**b**) Bar plots summarize the changes for SYN, Fast-SYN, and Lag contexts relative to the ALT condition, both in passive and behaviour conditions. All conditions led to large and significantly diverse effects, but only during behaviour (indicated by stars; SYN versus ALT: *P*<0.001; fast-SYN versus ALT: *P*<0.002; ALT and Lag contexts: *P*<0.010). Error bars indicate standard error.

Two further control experiments were carried out in a third animal to test if the STRF enhancement/suppression following SYN/ALT contexts strictly reflected the temporal alignment of the tones and not any other unintended parameter differences between the sequences. The analysis focused on the NEAR conditions, which had the largest effects (Fig. 4). In the first control, we equalized the rates of the SYN and ALT sequences by doubling the presentation rate of the SYN tones (Fig. 1c). Since the SYN tones remained temporally coherent regardless of the rates, we expected the STRF changes to remain unaltered, as validated by the data depicted in Fig. 5a (panels i and iv). Responses of 39 single-units were recorded in this experiment. As before, the SYN enhancements were significant compared to the ALT suppression (panel iii), but only during task engagement (SYN versus ALT: paired *t*-test: *P*<0.001, *n*=39; fast-SYN versus ALT: paired *t*-test: *P*<0.002, *n*=39).

We also examined how temporal delay between the tones (or equivalently their degree of coherence or incoherence) affected the patterns of plasticity. To do so, we compared STRF changes from the same cells and experimental sessions, but measured in the different contexts of SYN, ALT, and a time ‘Lag'. For the latter contexts, the tone sequences were moderately desynchronized by delaying the B-tones by 40 ms relative to A-tones (Fig. 1c). Such a time ‘Lagged' sequence is desynchronized midway between the completely coherent SYN and incoherent ALT. Figure 5 demonstrates that the STRFs adaptations tracked the same trend (n=23), changing from enhancement in SYN contexts (panel i), to no change in ‘Lag' (panel ii), to suppression for ALT (panel iii). Comparing the three STRFs during behaviour, there was no significant difference between SYN and Lag contexts (paired *t*-test: *P*<0.107, *n*=23), but a significant suppression between the ALT and Lag STRFs (paired *t*-test: *P*<0.010, *n*=23; although only marginally after a Bonferroni correction 0.05/7=0.007).

### Opposite correlational changes for SYN and ALT contexts

We next sought evidence of enhanced and suppressive interactions by assessing the spiking correlations among simultaneously recorded neurons as they responded to the SYN and ALT sequences. Specifically, based on the hypotheses of Fig. 1a, and STRF modulations in Figs 2, 3, 4, 5, we expected that when the animal listened attentively to the sounds (during the task), the distributions of pairwise spiking correlations (that is, between cells A and B in Fig. 1a) should trend in opposite directions for ALT versus SYN contexts reflecting the decreasing and increasing effective interaction, respectively.

**Figure 6:**
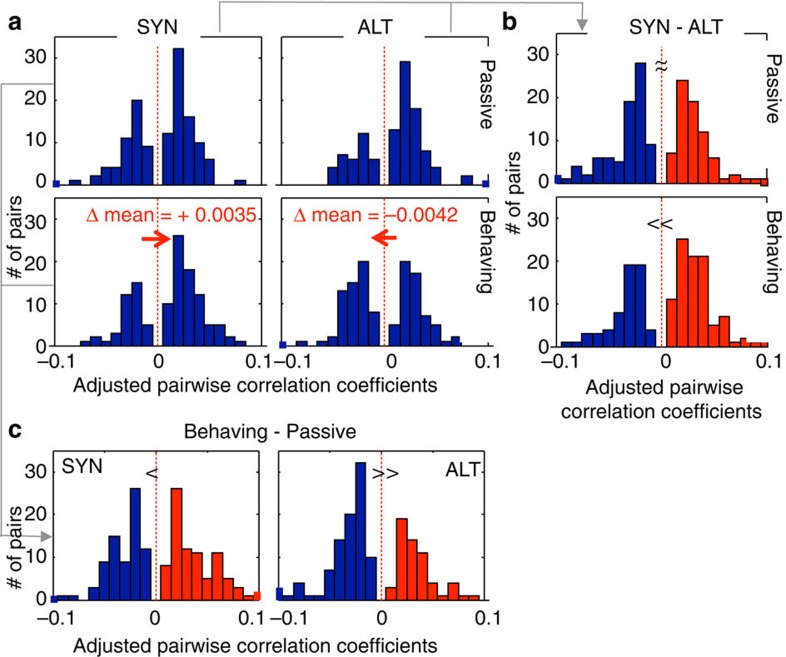
Distributions of adjusted pairwise spiking correlation coefficients in SYN and ALT contexts. (**a**) Adjusted correlation coefficients (that is, the correlation minus the same measurement after shuffling order of all trials) are shown during passive listening (top panels) and behaviour (bottom panels), in SYN and ALT contexts (left and right). Only non-zero correlation values (absolute value>0.01) were included in these displays, but no data were excluded for statistical analysis. The distributions following SYN and ALT contexts were similar in the passive state. During behaviour, they shift in *opposite* directions, with SYN becoming slightly more positive, and ALT more negative. (**b**) Distribution of differences of correlations between SYN and ALT conditions (SYN—ALT). Differences were approximately symmetrically distributed around zero during passive listening (top panel) and significantly biased towards positive values (*p*<0.004) during behaviour (bottom panel). (**c**) Distribution of differences of correlation (Behaviour—passive). Differences were significantly biased towards negative values during ALT contexts (*P*<0.014; right panel), but only marginally shifted towards positive values during SYN contexts.

The spiking correlations were computed for 256 simultaneously recorded response pairings (only responses from separate electrodes were included). The pairwise correlations were computed for each of four conditions: Passive SYN/ALT, and Behaving SYN/ALT. Within each condition, the pairwise correlation was computed as the average from a restricted set of stimulus conditions (namely only those stimulus combinations in which each neuron responded exclusively to one of the two tones; see Methods section for details). This minimized the contributions of shared inputs, although all correlations were also adjusted by subtracting correlations from trial-shuffled responses. The resulting distributions are shown in Fig. 6a. A two-way repeated-measures ANOVA showed a significant interaction between passive/behaving and SYN/ALT (*F*(1,510)=8.53, *P*<0.004, *n*=256) states and stimuli. During the passive state, the correlation distributions during SYN and ALT context are essentially the same (top panels in Fig. 6a). Fig. 6b (top panels) shows that the distribution of differences of adjusted correlations between SYN and ALT during the passive state are symmetric around zero, that is, showing no significant bias (paired *t*-test: *P<*0.225, *n*=256). This exactly as was found earlier for the target STRFs (Figs 2 and 3). However, the correlations during behaviour diverged slightly but significantly depending on the context (bottom panels in Fig. 6a). The bottom panel of Fig. 6b shows this significant divergence in the adjusted correlations between SYN and ALT (paired *t*-test: *P<*0.004), a manner that approximately mirrored the pattern of STRF changes seen earlier in Fig. 3. Hence, during behaviour, the adjusted correlations decreased significantly in the ALT sequences (passive versus behaving: paired *t*-test: *P<*0.014, *n*=256; Δ mean=−0.0042), while they trended (insignificantly) in the opposite direction for the SYN sequences (paired *t*-test: *P*<0.093, *n*=256; Δ mean=+0.0035). Strikingly, these changes vanished during the second passive session following task performance (paired *t*-test: *P*<0.771, *n*=256), consistent with the usual lack of rapid plasticity during passive sessions.

In summary, the interactions among responsive neurons (as reflected by their spiking correlations) rapidly adapted during attentive listening in a manner that was broadly consistent with the temporal coherence hypothesis (Fig. 6). The trends, however, were smaller and less significant than those seen in the STRF changes of Fig. 3, perhaps reflecting the very different nature of the measurements, as we shall discuss later.

### Average plasticity during the context sequences

**Figure 7:**
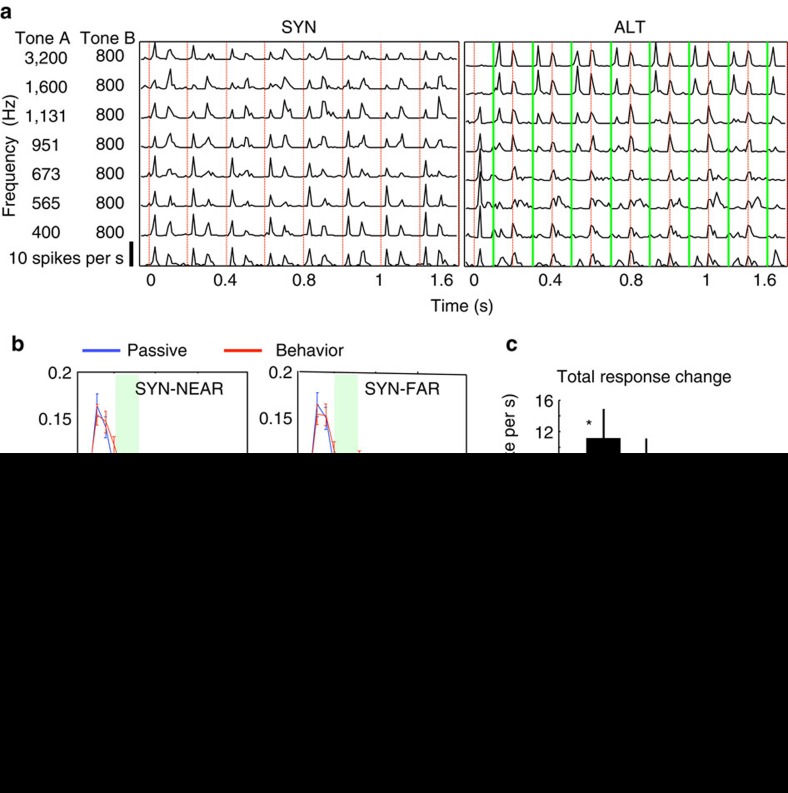
Task related plasticity effects in responses to the context tone sequences. (**a**): Example of PSTHs of responses to tone sequences of a neuron measured during passive SYN and ALT trials. Red and green vertical lines indicate the onset of the A and B tones, respectively, in both the SYN and ALT conditions. (**b**) Averaged period histograms measured during behaving and passive conditions. The horizontal black bars represent duration of A and B tones. Traces are the responses to the tone in the passive (blue curves) and behaving (red curves) conditions. Responses were measured in two windows: the total*-*response window (black bar) quantified the average response 0–80 ms after each tone onset; The green horizontal bar and shaded region mark the *sustained* response window (50–80 ms), that is, ignoring the tone onset responses. The upper panels are the average period histogram responses to the SYN sequences, in NEAR (left) and in FAR (right) conditions. Responses were enhanced during behaviour (red above blue traces). Lower panels show averaged period histogram responses obtained from ALT trials. ALT NEAR responses in the sustained window experienced significant reduction during behaviour. Error bars along the PSTHs indicate the standard error in each 10ms bin. (**c**) Summary of all changes in response amplitudes between behaving and passive states, with total response changes (black bars) and sustained response changes (green bars). In the total response window (0–80 ms), average responses to SYN-NEAR showed significant increase during behaviour (*P*<0.005). During the sustained response window (50–80 ms), responses to SYN significantly increased for both NEAR (*P*<0.001) and FAR (*P*<0.002) during behaviour. Responses to ALT-NEAR were significantly reduced (*P*<0.004). Error bars indicate standard error.

The divergent pattern of plasticity measured during and after the SYN and ALT sequences, and its complete absence during passive listening, suggest that the changes commence and build up during the context period when the animals are attentively listening to the SYN and ALT tones. To test this conjecture, we measured directly the dynamics of the response modulations as quantified by their post-stimulus and period histograms (PSTH). Examples for one unit are shown in Fig. 7a for two specific SYN and ALT sequences. A1 cells generally exhibited phase-locked responses to the tones. The strong response at the onset of the sequence rapidly decreased, and then fluctuated (on average) throughout the remainder of the trial. The analysis here focused on responses from 182 cells that were tuned within ±0.5 Oct of the fixed B tone-sequence, although still responsive to a wider range of frequencies. The period histogram of responses to the 3rd to 8th tones of each of four different conditions were computed as in the panels of Fig. 7b: SYN-NEAR (top-left), ALT-NEAR (bottom-left), SYN-FAR (top-right) and ALT-FAR (bottom-left), for both passive and behavioural conditions (blue/red curves).

We first consider the plasticity induced during the context. It is measured in terms of the average changes in the ‘total' response to each tone integrated over the 0–80 ms time-window (indicated by the black-bars in Fig. 7b). Such estimates were analysed by three-way repeated measure ANOVA, in which SYN/ALT and NEAR/FAR were treated as two factors and passive/behaviour was treated as a repeated measure. ANOVA indicated significant interaction between passive/behaviour and SYN/ALT (*F*(1, 724)=2.12, *P*<0.044, *n*=182). In the SYN condition (top panels), NEAR sequences exhibited significant response enhancements during behaviour (red versus blue curves, pair *t*-test: *P*<0.005, Wilcoxon signed-rank test: *P*<0.026; *n*=182), while FAR sequences followed the same trend, although not statistically significant (pair *t*-test: *P*<0.064, *n*=182). For the ALT sequences (bottom panels of Fig. 7b), we observed the anticipated suppression (or lack of enhancement) of responses during behaviour, although its strength depended on the response window over which the average was computed. This is because the ‘onset' responses during behaviour (0-50 ms) exhibited a phase-lead that countered the net suppression evident in the later ‘sustained' part of the responses. Consequently, averaging over the total response window (marked by the black bar in Fig. 7b) yielded no net suppression (Fig. 7c). Significant suppression, however, was readily seen in the responses (bottom panel of Fig. 7c; paired *t*-test: *P*<0.004, Wilcoxon signed-rank test: *P*<0.001; *n*=182) when measured for the sustained response window (50–80 ms, marked by the green bar and shading in Fig. 7b).

In summary, there was an enhancement of responses during the SYN compared with suppression for ALT contexts, and the suppressive effects were especially substantial for the sustained ALT responses. As we discuss in detail later, a likely explanation for the difference in the detailed nature of the adaptive effects during context versus post-context (STRFs) is the considerably different nature of the stimuli underlying the two types of measurements. The STRFs reflected responses to (target) tone clouds, which were random and overlapping, and hence tone onsets were not prominent.

### Dynamics of adaptation during the context

**Figure 8:**
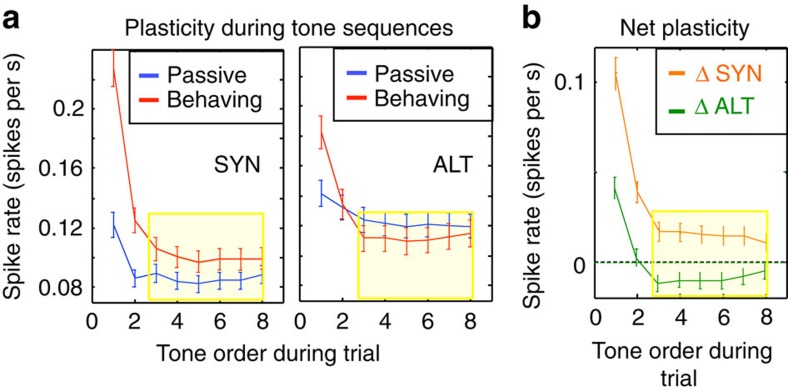
Dynamics of the rapid plasticity averaged across cells. (**a**) Plasticity of the averaged sustained responses to the tone sequences. SYN responses during behaviour (red curves in left panel) were enhanced at the onset, but then decayed rapidly (within three tones) to a higher level relative to the passive baseline responses (blue curves in left panel). For the ALT (right panel), the decay was more severe, with the responses becoming suppressed relative to the baseline (red curves below blue curves). Yellow shaded boxes indicate the times during which ALT and SYN responses reached steady state following the rapid decrease at the onset of the sequence. Error bars indicate standard error in the period of each tone. (**b**) Net plasticity during behaviour. Plot of the net plasticity during the context sequences. It is computed as the difference between the red and blue traces in the two panels of **a**. Dashed axis signifies the baseline passive response for the SYN or ALT sequences. Orange trace is the net enhancement of the SYN responses during behaviour. Green trace is the net suppression relative to the passive baseline of the responses to the ALT sequences. Error bars indicate standard error in the period of each tone.

So far, we have estimated the average change during and after the context tones, rather than the dynamics of the change as it occurred. Here we examine how rapidly the responses become enhanced or suppressed during the first few pairs of tones in a context sequence. We focused on the trends in the sustained tone responses (Fig. 7b) because of their larger sensitivity (Fig. 7c). Figure 8a illustrates the dynamics of this plasticity, specifically how responses to SYN and ALT sequences in the NEAR condition rapidly adapted during the passive and behavioural conditions (blue and red curves, respectively). In both cases, the response to the first tone pair (the sustained response in one period) in the sequence was largest, and became even larger during behaviour. Responses to subsequent tone pairs rapidly decreased to a flat level within three tone pairs (highlighted within the yellow shaded box). For the SYN sequences (Fig. 8a; left panel), the responses reached a steady state that was significantly higher (enhanced) relative to the passive responses (red above blue curves). For the ALT sequences (Fig. 8a; right panel), the opposite occurred; responses during behaviour became suppressed relative to the passive (red below blue curves). To highlight these patterns, we plotted in Fig. 8b the changes explicitly, emphasizing that the adapted responses to SYN and ALT sequences maintained their dynamics, sign, and significance throughout the trial.

Finally, it is important to emphasize that the contrasting response rates and correlations and STRF changes shown in all these figures were averaged from many randomly interleaved trials of SYN and ALT sequences. This means that the enhancements (in SYN) and suppression (in ALT) remained distinct and did not cancel out by the averaging across all trials, and hence must have developed rapidly on a trial-by-trial bases. In fact, it is evident from Fig. 8 that the adaptive effects of temporal coherence built up rapidly within three presentations of the tone pairs, that is, at about 400 ms from the onset of the sequence. This duration is comparable to the time-course of the build up of streaming in ALT sequences reported in human psychoacoustics^20^,^21^,^22^.

## Discussion

This study investigated the physiological evidence for temporal coherence computations, and the rapid adaptive processes that potentially play an important role in auditory scene analysis, and more generally in source segregation and segmentation in auditory, visual, and other sensory systems. In many psychoacoustic studies of streaming and scene analysis, the behavioural tasks depend on a subject's ‘introspection' to report whether the percept is that of a segregated or an integrated stimulus^18^,^23^. This approach is clearly infeasible in animal experiments where instead one has to rely on objective behavioural measures that indirectly assess streaming, for example, by the animal's ability to detect deviants in a target stream while avoiding interference from another simultaneous stream^19^. However, as explained earlier, to avoid the confound of enhancements induced by selective attention to one stream^16^,^24^, we have opted for a global attention paradigm to show that temporal coherence of stimuli leads to effects that may play a key role in the perception of streaming.

Our observations also suggest that attention is necessary for this rapid plasticity. In an earlier neurophysiological and psychoacoustic study of the perceptual segregation of tone sequences^16^, we found no evidence in the neuronal responses in primary auditory cortex of the passive awake animal that could explain the dramatic perceptual difference between ALT and SYN tone sequences. Specifically, in both cases, driven neurons exhibited the same tuning curves and other properties regardless of the temporal coherence between their responses. Consequently, we speculated that when an animal actively attends to the stimuli in a behavioural setting, an adaptive process is initiated that induces interactions and response changes that differentiate the two percepts, as described in the schematic of Fig. 1.

This conjecture was directly tested in this study in three ways. First, during attentive listening, STRF measurements immediately following the temporally coherent and incoherent sequences diverged significantly (Fig. 3). This difference did not exist during passive listening, and it was likely due both to enhancement and suppression processes following the SYN and ALT contexts, respectively (Fig. 3).

The second line of evidence concerns plasticity during the responses to the ALT and SYN tone sequences. Specifically, response amplitude became increasingly more suppressed in ALT sequences, while responses to SYN tones were enhanced (Fig. 7), a divergence that was both rapid and significant, taking no more than 2–3 tones (<0.5 s) to achieve its final level (Fig. 8). Finally, these rapid adaptations were also paralleled by changes in the spiking correlations between pairs of neurons that were activated separately by the ALT and SYN two-tone sequences (Fig. 6). It was, however, impossible to determine the dynamics of the spiking correlations during the context because of the small number of spikes available to compute them.

The rapid dynamics of this plasticity explain why significant changes were measurable during and following the two contexts despite the fact that the SYN and ALT trials were completely interleaved throughout the behavioural task. Without this rapid buildup of strong and reversible adaptive effects, averaging the results would have washed away the opposite changes.

Finally, we emphasize that the STRF and response modulations described here cannot be attributed to ‘simple (artifactual) effects' such as adaptation to the faster tone rates of the ALT compared to the SYN sequences, since doubling the latters' rates induced the same enhancement (Fig. 5). Neither did the interactions between the responses of the two sequences play a significant role since plasticity patterns were the same when spiking correlations were computed from responses to well-separated tones, that is only those stimulus pairs in which each neuron responded exclusively to one of the two tones (Fig. 6).

Plasticity also depended on the stimulus spectrum. Specifically, rapid STRF plasticity, and response changes to tone sequences were stronger when the tones were more closely spaced (NEAR), becoming weaker but still significant when the spectral separation between the tones was large (FAR), consistent with psychoacoustic findings^2^. The weak dependency on spectral distance, and the persistence of the effects even for separations of over an octave, implies that interaction between neurons at proximate frequency channels was not the only mechanism underlying the rapid plasticity. Segregation and binding occur over large spectral distances, depending on the context of the stimuli. Furthermore, binding may act on multimodal features (e.g., auditory and visual) and hence presumably over even longer distances.

There are numerous postulated mechanisms that may underlie the observed rapid plasticity. Conceptually, the changes are analogous to a fast version of ‘Hebb's rule' of associative connectivity in which neurons ‘wire together if they fire together'. The fast dynamics we observe however suggest that it is more likely to be a short-term modulation of pre-existing (silent?) synapses due to correlated activity. It is known that synaptic strength can be rapidly and profoundly modulated within milliseconds after the onset of specific temporal patterns of activity^25^,^26^,^27^. These changes can be facilitative or depressive, and may alter profoundly the nature of the information processing in a neural network^28^,^29^. An alternative mechanism might be top-down modulation from outside primary auditory cortex. Further experiments with recordings in secondary auditory cortex and prefrontal cortex may help clarify this issue. Alternatively, the most direct view of the changing connectivity might be captured by SYN and ALT pulsatile stimulation of cortical cells *in vitro* slices. Apparently, such a straightforward experiment has not yet been done.

The patterns of rapid plasticity in the present experiments strongly resemble those previously described in auditory, visual and motor cortical responses^17^,^30^,^31^,^32^,^33^,^34^,^35^,^36^. The one marked difference is the short-time persistence of the plasticity effects in the STRFs following the end of the trial (Fig. 3e). This is sensible if the plasticity effects are designed to evolve rapidly to track different sources as one shifts the attentional focus, and hence groups feature channels differently. In our previous experiments^17^, the attentional focus was maintained for much longer (20–30 min) than a single trial (∼2–5 s) and hence task-related plasticity effects may have had time to deepen, and hence persist longer after the task.

A curious aspect of the enhanced and suppressed responses in these experiments is that they became more prominent about 50 ms after the onset of a tone response, a time-window we generally referred to as the ‘sustained portion' of the response (Fig. 7). In humans, this is approximately the relative time delay necessary to render two tones perceptually desynchronized^37^,^38^,^39^. This delay may reflect the multi-synaptic nature of the adapted mutual connectivity, or perhaps the complex nature of the adaptive synapses, which require time to integrate their inputs before inducing their effects^40^. Whatever the underlying causes, it is evident that as temporal coherence gradually decreased with the desynchronization of the tones, the enhancement gradually gave way to suppression as demonstrated by the lagged tone sequences (Fig. 5).

It should be emphasized that natural stimuli have far more complex spectrotemporal structure than the precise repeated onsets of the tone sequences. For example, in speech mixtures, frequency channels driven by different speakers are more likely to be incoherently driven by random and sometimes overlapping activation patterns similar to the incoherent activations of different frequency channels in the tone clouds^41^,^42^. Similarly, coherently (and repeatedly) activated channels due to a single speaker are rarely as perfectly lined up as in the SYN tone sequences^43^. Consequently, the overall suppressive and facilitatory influences would probably be more sustained and effective among channels with a mixture of such randomly driven incoherent and coherent responses than seen for short perfectly alternating or synchronized tone sequences.

The measurements used here to detect the adaptive suppression and enhancement are quite diverse in nature. They included STRFs with target tone clouds (Figs 2 and 3), spiking correlations (Fig. 6), response period and PST histograms (Fig. 7), and sequences of simple tones (Fig. 8). As is well appreciated, neuronal stimulus-response characteristics are fundamentally nonlinear and hence characterizing them, be it through STRFs, frequency-tuning curves, gain or any other measure, depends on the exact nature of the stimuli used^44^,^45^. This fact may explain why SYN and ALT contexts displayed different degrees of enhancement and suppression depending on the way they were measured. Consequently, it is more important to realize that regardless of how measurements were done, SYN and ALT plasticity always maintained the same relative sense (or sign), but exhibited more suppression in some ALT contexts (for example, Figs 2 and 3), or more enhancement in other SYN contexts (for example, Figs 7 and 8).

The role of selective attention in streaming becomes now evident in light of the rapid induction of mutual connectivity according to the temporal pattern of driven responses discussed earlier. Specifically, in the ALT context, the connectivity pattern is mutually competitive with neurons suppressing each other. However, selective attention to one source enhances its representation and suppresses effectively other uncorrelated sources (or distractors). By contrast, when the context is coherent as in the SYN sequences, the induced mutual connectivity is cooperative (excitatory), and hence we predict that attending selectively to one tone should have the opposite effect on the neighbors, that is, enhance their responses and effectively ‘bind' them to the attended response.

Note that in realistic scenarios such as speech mixtures, both coherent and incoherent responses coexist in the responses reflecting features that belong to the same source (coherent) and competing sources (incoherent). Thus, when attending to one feature, it not only suppresses all other temporally incoherent features, but it also binds the attended feature to all other coherent features (that belong to the same source). This is presumably how attention to one feature such as the timbre, location, or pitch of the voice of one speaker can help its segregation from that of other speakers in a mixture^46^, and simultaneously group the target features with others that are not explicitly attended to. This is how, we believe, the sensation of an auditory source (object) emerges. This hypothesized scheme has been successfully implemented in a computational algorithm recently to demonstrate its effectiveness in complex sound segregation^42^.

Finally, temporal coherence is conceptually applicable to all sensory and motor responses regardless of their origin. In vision, an object moving in the environment has all its edges, corners, textured surfaces and coloured and uncoloured pixels move in the same direction and speed, inducing coherent responses in various visual cortical areas. By contrast, the responses induced by objects moving independently of each other are presumably temporally incoherent. Consequently, the implications of all the findings reported in this study apply equally to such visual responses, thus explaining how visual objects become perceptually segregated^47^ and how these ideas from viewing motion of rigid bodies can in turn be exploited effectively to segregate auditory streams^48^. Multimodal percepts can be equally well explained by temporal coherence. For example, lip-reading is a well-known contributor to speech comprehension, especially in noise^49^. Here the visual signal (lip-opening) is coherently modulated with the power in the speech signal and its features, and hence can serve as a strong binding anchor for the speech signal as a whole^42^.

## Methods

### Behavioural task

All experimental procedures were approved by the University of Maryland Animal Care and Use Committee and conformed to standards specified by National Institutes of Health. Two adult female ferrets were trained to listen to a sequence of tone pairs (reference) followed by random cloud of tone pips (target). They were required to withhold licking during the reference but then to lick during the target (0.1 s after its onset) to receive a water reward (0.1–0.15 ml). Any lick during the reference or before 0.1 s following target onset resulted in immediate termination of the trial as well as a 4 s timeout. Behavioural performance was quantified as percent of correct trials in each behavioural session. A trial was labelled incorrect when the animal licked the waterspout during the tone sequence (false alarm) or did not lick the waterspout during the target (miss). For the data analysis, only the correct trials were used. During electrophysiology recordings, the identical set of stimuli was presented while animal was passively listening before and after the task performance. In all experiments, animals were trained to just listen to the sounds if there was no water reward. They were also trained expect to a task to begin when an LED light is turned on, and additionally a few drops of water form on the waterspout. The animals usually lick them, the waterspout then turns off, and the task begins. All stimuli were presented at 60 dB SPL.

### Surgery

To secure stability for electrophysiological recording, a stainless steel head-post was surgically implanted on the skull. Ferrets were anaesthetized with a combination of Ketamine-Dexmedetomidine for induction and isoflurane (1–2%) for maintenance of deep anaesthesia throughout the surgery. The skull was surgically exposed and the head-post was mounted with dental cement, leaving clear access to primary auditory cortex in both hemispheres. Antibiotics and post-surgery analgesics were administered as needed after surgery.

### Neurophysiological recordings

Experiments were conducted in a double-walled sound attenuation chamber. Small craniotomies (3-5 mm diameter) were made over primary auditory cortex before recording sessions. Responses were made with four tungsten microelectrodes (FHC, 3–8 MΩs) advanced independently by a multielectrode manipulator (Alpha-Omega). Electrode signals were amplified and filtered (bandpass 0.3–6 kHz), then acquired at 25 kHz. Pure tones (0.25–32 kHz, 150 ms duration) were presented to search for responsive sites, until single-unit responses with good isolation were found on majority of the electrodes. Single unit (one to two neurons per electrode) were sorted off-line by *k*-means clustering using custom MATLAB software.

### Stimuli

Tone sequence consisted of two frequencies denoted as A and B (Fig. 1), 75 ms in duration, 5 ms cosine ramp, with 125 ms gap between tones in the same sequence. The A and B sequences were presented either synchronously (SYN) or alternately (ALT), randomly selected across trials. In each behavioural session, the frequency of B sequence was fixed, while the frequency of the A sequence changed randomly at 24, 12, 6 and 3 semitones away from B tones. The length of the tone sequence varied randomly between 1.6–4.8 s. The random cloud tone pips consisted of 64 tones (25 ms duration, 5 ms cosine ramp). Frequencies of the tones were chosen from 64 frequency bands in a five-octave range around the frequency of B tone with one semitone steps. All tones were presented at random times during a 1.6 s long tone cloud. All tones were typically played at 60 dB SPL. The loudness of the tone pairs, however, might vary depending on the absolute frequency of each tone and the frequency separation between them. We did not control for these variations because we felt that the loudness effects could not systematically bias the results in any particular direction since we accumulated data from a large number of cells, and in paradigms with a large number of frequency combinations.

### Data analyses

Neurons' tuning properties were measured by neural responses to 660 tones with randomly varying frequency and intensity (0.25–32 kHz, 20–70 dB SPL, 150 ms duration, 1.05 s inter-tone interval). Tuning curves of neurons were extrapolated along the frequency-intensity contour lines, where responses were at least two standard deviation higher than the baseline spike rates. Characteristic frequency was taken as the frequency at the lowest intensity on the tuning curve. STRFs (Figs 2, 3, 4) were calculated as the cross correlation between onsets of the tones on each frequency band and the corresponding spike train for the given trial and then averaged across trials. For analysis, all STRFs were smoothed by a 3 × 10 filter. Only neurons with STRFs of high signal-to-noise ratios >0.3 were analysed^17^. To average the STRF across neurons, they were aligned together by their center frequencies. For convenience of alignment, 23 frequency channels at the low and high frequency margins of the STRFs were not used.

Differences between SYN and ALT were firstly analysed in each pixel of STRF (one semitone and 1 ms bin) by paired *t*-tests. The significance level was adjusted by the Bonferroni correction (*P*<0.00001). Contours were then drawn around the regions that showed significant differences between SYN and ALT. Then, we calculated the peak amplitude for each STRF, defined as the averaged response value between 20 and 70 ms (following stimulus onset) and 20 semitones around the CF of each neuron. Finally, STRF peak amplitudes for SYN and ALT were analysed across all neurons.

To calculate the spiking correlations between neurons, we (1) selected neuron pairs that they responded to one of the two-tone sequences (that is, their responses are segregated). The standard to make this selection is that the neuron must have more than twice the spike rate (measured over an interval of 100 ms after each tone's onset) elicited by one tone than by the other tone in an alternating tone sequence. (2) Then for each selected neuron, we extracted spike timing from all recorded trials with a 2 ms bin, allowing 1ms jitter for calculation of spike train correlation. Only the spike timings of spikes that occurred in the first 8 tone pairs in each tone sequence were used. (3) For each neuron pair, the cross-correlation between the spike trains from all simultaneously recorded trials was calculated, following the procedure reported in Atencio and Schreiner^50^, with 1-50 ms time-shifts, the maximum of which was selected. (4) We similarly calculated the correlations between shuffled trials from same pairs of neurons, which reflected the correlation due to common inputs (stimuli) rather than to mutual interaction. (5) The correlation coefficients from the two previous measurements were subtracted to estimate the spiking correlation due to connectivity between the two neurons while removing the effects of a common input. These are referred to as the ‘adjusted correlations' as plotted in Fig. 6.

To compare responses to tone sequences in different contexts, the averaged period histograms (Fig. 7) for each context were calculated as follows: First, for a given neuron, the post-stimulus histograms of responses in each context type were calculated (average across trials, 10 ms bins). Period histograms were then computed by folding the post-stimulus histograms across each 200 ms period (first two periods in the sequence were excluded). Finally, for each context type, the period histograms from all neurons were averaged. Spike rates were first analysed in ANOVAs and paired t-tests and then confirmed with Wilcoxon signed-rank tests to avoid bias due to influence by those neurons with highest firing rates.

### Data availability

Data are available on request due to privacy or other restrictions.

## Additional information

**How to cite this article:** Lu, K. *et al*. Temporal coherence structure rapidly shapes neuronal interactions. *Nat. Commun.* **8,** 13900 doi: 10.1038/ncomms13900 (2017).

**Publisher's note:** Springer Nature remains neutral with regard to jurisdictional claims in published maps and institutional affiliations.

## Supplementary Material

Peer Review
