# Supplementary material for: Temporal coherence structure rapidly shapes neuronal interactions
Source: Nat Commun. 2017 Jan 5;8:13900. doi: 10.1038/ncomms13900 (PMC5228385; doi:10.1038/ncomms13900)
Supplement: Peer Review [file ncomms13900-s1.pdf]

PEER REVIEW FILE

Reviewers' comments:

Reviewer #1 (Remarks to the Author):

SUMMARY:

In this study, the authors test whether sound responses of neurons in the primary auditory cortex of ferrets are influenced by the temporal coherence of preceding stimuli. From their data, the authors conclude that this effect is observed when animals are engaged in a task but not when passively listening. They find that when the preceding stimuli are tones of different frequency presented simultaneously, neural responses and correlations across neurons are larger than when preceding stimuli are alternating tones.

COMMENTS:

The manuscript presents an intriguing set of observations regarding the effects of temporal coherence on neural responses. However, as explained below, several issues in methodology and interpretation challenge the validity of the conclusions.

1. The use of the term "neuronal connectivity" in the context of these experiments does not seem appropriate. The paper emphasizes this term throughout (including the title), but the measurements only reflect correlations not actual connectivity. The observed changes in STRFs and neural correlation could be the result of changes outside the primary auditory cortex (without changes in connectivity in the auditory cortex).

2. The authors propose a model (Fig.1A) in which simultaneous tone sequences strengthen the connections between neurons with different frequency tuning. From this model, one would predict that the effect of SYN tone sequences is an enhancement of responses to frequencies outside the preferred frequency of the neuron being measured. Similarly, ALT sequences would inhibit responses outside the preferred frequency. However, the main effect seems to be an increase/decrease in the peak response of each neuron.

3. The comparison of sound-evoked responses between passive and behaving conditions does not seem appropriately controlled. Measurements during the behaving condition are done while animals are licking a water spout, and therefore, a few factors not accounted for by the authors

could explain some of the changes in neural activity between passive and active conditions. These include:
a) Effects of reward on the responses of auditory cortical neurons.
b) Sounds produced by licking.
c) Motor signals feeding back to AC related to licking.

4. The authors should address the possibility that motor responses of the ferret are entrained by the preceding sequence of tones. This could result in differences between the SYN and the ALT conditions (via the mechanisms presented in the previous point).

5. The methods section does not explain clearly the difference between the passive and behavior conditions. Was there any indication that animals knew if they were in the passive or task conditions? The results section mentions briefly an LED, but nothing else is stated.

6. The methods section states how performance is quantified, but results are never shown. It is also not stated how these performance levels are used.

7. Were only correct trials used in the analysis of neural responses? This should be stated in the methods.

8. Averaging firing rates across neurons (as in Fig.7B) can be misleading. The result will be largely influenced by those neurons with highest firing rates. The authors would have to demonstrate that

9. Why were correlations calculated only from a restricted set of stimulus conditions?

10. When estimating spike correlations, it is good practice to use signals measured from distinct electrodes to avoid artifacts due to imperfect unit isolation. The authors should explain if the measurements come from the same or different electrodes and make sure unit isolation does not introduce correlation artifacts.

11. Frequency is expressed in octaves in the text, but in semitones in the figures. This makes it harder to relate these quantities (for example the frequency of different tones with respect to the response in the STRFs).

12. The figures need to indicate significance levels (p-values) whenever a star is used to indicate significance (see for example, Fig.5B).

13. All figures showing STRFs need to have colorbars (see Fig.2), and these colorbars need to have units.

MINOR COMMENTS:

- In Fig.7C, what is zero in the time axis? sounds do not seem to start at zero.

- In lines 423-424, it is not clear what the authors mean by "computed from responses to completely segregated responses".

- Line 599 says that several intensities were used, but line 600 does not say which intensity was used for calculating best frequency.

- It would be easier for the reader is panels in Fig.1C where located in Fig.5 where the results are presented.

- Line 200: color black dots? The authors probably meant orange stars.

- Line 300 says that the PSTH was calculated for 4 conditions, but it is not clear from the figure which plots correspond to each condition.

- The vertical axis in each inset in Figure 7A needs to be defined.

- (Line 133-134) Saying plus/minus and above/below is redundant.

- (Line 145) Typo: "the how"

- (Line 270) Close parenthesis.

- (Line 295) the words do not correspond to the meaning of PSTH.

- (Line 775) Typo: missing words.

Reviewer #2 (Remarks to the Author):

The authors provide compelling evidence for rapid changes in the correlational structure and response properties of neurons in the primary auditory cortex (A1) of behaving ferrets as a function of the temporal coherence and spectral properties of acoustic stimuli presented during the recording. In particular, they find that firing rates, responsiveness, and interneuron spike-to-

spike correlations were all rapidly enhanced by synchronous stimuli, whereas alternating streams of tones suppressed these aspects of the neural responses. Importantly, they show that these effects only occur when the animals were engaged in the task and presumably attentive to the stimuli.

I found this to be an interesting study on an important topic in auditory processing that relates to the role of temporal coherence in the perception of auditory streams. The results are consistent with the perception of single vs. pairs of auditory streams for human listeners as a function of the repetition rate, the temporal synchrony or alternation of the two sets of tone sequences, and the separation in frequency between the two tone sequences. I found the Discussion section to be insightful and I appreciate the various predictions the authors make based on their experimental findings and interpretation. I believe that the experimental and theoretical methods are all appropriate, including the controls, and I think this paper will be of interest to a wide readership.

I have some questions and comments for the authors concerning the interpretation, presentation, and clarity of some parts of the manuscript.

Specific comments:

The authors interpret the changes they measure in correlations between neurons in terms of the connectivity in the network. This is not unreasonable, but I do not believe they actually provide specific evidence for changes in connection strengths between neurons, since these changes in correlation could be due to changes in the dynamics of the neural activity (e.g., changes in synchrony of presynaptic spikes arriving at synapses of the recorded neuron) even if the synaptic strengths themselves are not changing. They point out that there are reports in the literature of changes in synaptic strength in A1due to short term synaptic plasticity, measured using patch clamp methods, and I agree with them that this is likely to be occurring here, but I think they should make it clear that their results do not prove this; rather, they are consistent with this.

Line 352:
"...and hence must have developed rapidly during each trial." I agree that the effect developed rapidly, but I do not think that the average response guarantees that the effect was present in "each" trial.

Fig. 3C and D:
These scatterplots would be slightly easier for me to view if they were square in shape so the horizontal and vertical scales matched, and if they had thin diagonal lines to indicate equality.

Fig. 4 caption:
It took me a minute to see that all plots in the figure are averages across the neural population, so

I would add "across all cells" or words to that effect in the figure title, similar to the caption to Fig. 3.

Fig. 4:
It is hard for me to see differences in these plots. It would help if numbers appeared in the corner of each plot (as in Fig. 2) or if color plots of differences were included (as in Fig. 3B).

Fig. 6B caption, line 934:
I believe "proximately" should be "approximately" here.

Fig. 6:
I do not understand Fig. 6B and C. The caption states that these are differences in the correlations, not correlations of the differences; is that correct? What exactly is being plotted? I would have expected the histograms span a smaller horizontal range than the full range spanned by the original correlation histograms.

Fig. 7A caption, line 944:
The caption mentions red and green vertical lines, but I see no color in Fig. 7A.

Line 121:
I think "...it would have resulted in..." should use "might" or "could" here.

Line 145:
"To assess the how" -> "To assess how"

Reviewer #3 (Remarks to the Author):

This is well written manuscript that describes a series of experiments on the temporal coherence and its relevance to auditory stream segregation. The experiments were cleverly designed and the results are interesting. That being said, there are some problems with the terminology used and the citations in the text are not always accurate.

Better defining some of the terms and concepts would strengthen the manuscript. For instance, in the text, rapid changes in neural firing appear to be equated with rapid plasticity. But they are not necessarily the same thing. For instance, changes in neural firing rate could also index attention and/or arousal associated with task demands. It is unclear how one could distinguish between these alternative possibilities. The authors should consider providing an example of a rapid neuroplastic change in the brain or adding a list of prerequisites used to conclude that changes in

firing rate index rapid neuroplastic changes rather than rather than attention or arousal. In the abstract (line 27), there is a distinction made between perceptual streams and sources, "features are segregated into different perceptual stream and sources." The authors should clarify what is the difference.

The title of the manuscript leads the reader to expect analyses that focus primarily on functionally connectivity. However, the analyses that was done focuses primarily on the strength of the responses, as opposed to actual changes in correlations between units. I suggest that the correlation analyses be moved up in the results section because it is the novel element of the study. Moreover, I think that including more detail regarding the correlation analyses is needed and will improve the paper. For instance, Figure 1 suggests some directionality, but it is unclear (to this reviewer) how direction can be inferred from the correlation analysis.

In the present study, frequency separation between the two tones that composed the sequence is manipulated. Increased frequency may induce changes in loudness perception. This should be discussed. Also, it is unclear how changing the frequency separation between two tones presented simultaneously can inform us about temporal coherence. For instance, line 227 mentions that temporal coherence of the stimuli gradually decreased as the stimuli became more separated in frequency. How could this be since the timing does not change?

Line 40, the statement "the physiological mechanisms that underlie this ability remain unknown' is too strong and does not sufficiently acknowledge more than 20 years of research on this topic. Please revise.

Line 42, I would replace "perceptual" with "acoustic."

Line 50, perhaps I am misunderstanding, but I was under the impression that sequences of synchronous tones do segregate when the frequency separation between the two tones is large (e.g., one octave separation). This is also what is shown in Elhilali et al. (Neuron, 2009).

Line 145, replace "To assess the how" with "To assess how."

Line 354-356, this sentence need to be revised. In humans, the build up of auditory stream segregation usually takes several seconds. To my knowledge, there is little evidence supporting the notion that temporal coherence occurs within 400 ms in human listeners. The references provided by the authors are inaccurate. It is unclear where in Bregman's book the authors found support for a build up of stream segregation in 400 ms. Also, unless I am mistaken, the study from Thompson et al. did not show build up in 400 ms but instead showed build up over several seconds. Please revise, clarify, and/or review carefully that cited work is appropriate (in this section but throughout as well).

Line 359-361, the sentence is awkward. I would omit "and the rapid adaptive processes that they."

Line 380, the reference is appropriate for MEG but not EEG. That study focused exclusively on MEG data. For an example of a study examining the effect of attention on EEG during stream segregation using ABA pattern, Snyder et al. (2006) would be more relevant and appropriate.

Line 472, please add the year after Turgeon et al.

Line 530, it is not clear what the difference is between voice and pitch. The ability to focus attention on a particular talker is likely driven primarily by its pitch. Or does voice include other features? Please clarify.

Line 733, reference 27, please correct the spelling of the second author. It should be Micheyl.

Line 813, please check reference 51, 2016 or in press?

Figure 2, it is unclear what the color coding scale is referring to. Are the panels on the same scale?

Figure 3, I found the figure difficult to understand. Please provide the units for the color bar.

UNIVERSITY OF MARYLAND

Shihab A. Shamma, Professor
Institute for Systems Research
Electrical and Computer Engineering
University of Maryland College Park
email: sas@umd.edu, Tel: (301) 405-6842

July 26th, 2016

Dear Sir or Madam,

Enclosed is the revised version of the manuscript entitled "Temporal Coherence Structure Rapidly Shapes Neuronal Interactions". We have examined carefully all the referees' comments, and made the recommended changes. This cover letter serves to explain exactly what changes we made.

Reviewer #1:
In this study, the authors test whether sound responses of neurons in the primary auditory cortex of ferrets are influenced by the temporal coherence of preceding stimuli. From their data, the authors conclude that this effect is observed when animals are engaged in a task but not when passively listening. They find that when the preceding stimuli are tones of different frequency presented simultaneously, neural responses and correlations across neurons are larger than when preceding stimuli are alternating tones.

COMMENTS:
The manuscript presents an intriguing set of observations regarding the effects of temporal coherence on neural responses. However, as explained below, several issues in methodology and interpretation challenge the validity of the conclusions.

1. The use of the term "neuronal connectivity" in the context of these experiments does not seem appropriate. The paper emphasizes this term throughout (including the title), but the measurements only reflect correlations not actual connectivity. The observed changes in STRFs and neural correlation could be the result of changes outside the primary auditory cortex (without changes in connectivity in the auditory cortex).

The reviewer is correct in that we do not explicitly measure actual changes in connectivity among neurons, but rather neural correlates of that. So we have changed all such references in the manuscript (including the title) to say "interactions" or "presumed connectivity" or similar statements, rather than "connectivity", to indicate these *indirect* measures. The only places where "connectivity" remained are where we discuss the original hypothesis of Figure 1. We also added a mention of the possible influences from outside of the primary auditory cortex in the discussion.

2. The authors propose a model (Fig.1A) in which simultaneous tone sequences strengthen the connections between neurons with different frequency tuning. From this model, one would predict that the effect of SYN tone sequences is an enhancement of responses to frequencies outside the preferred frequency of the neuron being measured. Similarly, ALT sequences would inhibit responses outside the preferred frequency. However, the main effect seems to be an increase/decrease in the peak response of each neuron.

The reviewer's prediction is accurate as it applies to the STRF of one neuron driven by a context of only two tones, *one* at CF and the other off the CF. To ensure getting effective changes, the experiments here presented many tones around the CF of a neuron in one block. So the effects on the STRF of one neuron were expected to spread out and not be localized at a single frequency. Furthermore, the STRFs shown in Figure 3 were averages from many (> 100) neurons, and these

come in a wide variety of shapes and sizes, and so the average is expected to emphasize the excitatory red region at the center, which is apparently enhanced after SYN sequences, and suppressed after ALT sequences.

3. The comparison of sound-evoked responses between passive and behaving conditions does not seem appropriately controlled. Measurements during the behaving condition are done while animals are licking a water spout, and therefore, a few factors not accounted for by the authors could explain some of the changes in neural activity between passive and active conditions. These include:
a) Effects of reward on the responses of auditory cortical neurons.
b) Sounds produced by licking.
c) Motor signals feeding back to AC related to licking.

We agree with the reviewer that the factors s/he mentions (a-c) all affect responsiveness of neurons in the cortex, as has been amply demonstrated in the literature. However, for our experiments, there are two important considerations that mitigate this concern. One is that our main result concerns the comparison between the two different contexts, SYN and ALT, *during* behavior. And for this set of results, all the factors mentioned by the reviewer apply equally to the two contexts, with absolutely no differences. Secondly, the comparison between the passive/behaving states simply shows that there were no differences observed between SYN and ALT in the passive state. However, while we agree that arousal and all other factors (a,b,c enumerated above) during behavior may affect responses, it is difficult for us to imagine how they could contribute so precisely and selectively to produce the range of effects seen. For these reasons, we do not believe that these factors can confound the main results.

4. The authors should address the possibility that motor responses of the ferret are entrained by the preceding sequence of tones. This could result in differences between the SYN and the ALT conditions (via the mechanisms presented in the previous point).

We are unclear of the meaning of this point. The animals were *not* licking water during the preceding sequences; they were expected to hold off touching the spout until the target cloud of tones arrived. So there were no "motor-related" differences at all between the two contexts. In addition, we have a fast-SYN condition in which synchronous tones presented at the same rate as the alternating tones, but it still showed the same effect as SYN. Thus, the rhythm of tones cannot explain the divergent effects that we see in SYN and ALT.

5. The methods section does not explain clearly the difference between the passive and behavior conditions. Was there any indication that animals knew if they were in the passive or task conditions? The results section mentions briefly an LED, but nothing else is stated.

We have added more clarifications in the Methods section (end of section on Behavior) on how the animals are trained to recognize the two states. Thank you for pointing this out.

6. The methods section states how performance is quantified, but results are never shown. It is also not stated how these performance levels are used.

In **Methods** (section on Behavior) we give the following: "Behavioral performance was quantified as percent of correct trials in each behavioral session. A trial was labeled incorrect when the animal licked the waterspout during the tone sequence (false alarm) or did not lick the waterspout during the target (miss). For the data analysis, only the correct trials were used. ". We also gave performance results during experiments in the 2nd paragraph of the section "Context influences neuronal STRFs during task engagement". Performance was quite stable throughout (with a mean

of 74% and standard error of 1%) so we felt it unnecessary to give more details or make a plot of the behavioral results.

7. Were only correct trials used in the analysis of neural responses? This should be stated in the methods.

Yes. This was stated in the **Methods** (section on Behavior): "For the data analysis, only the correct trials were used."

8. Averaging firing rates across neurons (as in Fig.7B) can be misleading. The result will be largely influenced by those neurons with highest firing rates. The authors would have to demonstrate that.

Indeed this is a possibility. We used Wilcoxon signed-rank tests (the non-parametric version of paired t-test) to re-evaluate the results and obtained the same significant results: In the window 0-80ms, SYN-NEAR showed significant differences between passive and behavior ($p = 0.0261$). In the window 50-80ms, both SYN-NEAR ($p < 0.001$) and SYN-FAR ($p = 0.0087$) showed a significant increase during behavior. ALT-NEAR showed a significant reduction during behavior in the same window ($p<0.001$).

9. Why were correlations calculated only from a restricted set of stimulus conditions?

Clarified in the text. Thank you for pointing this out. We have computed the correlations from well-separated responses to minimize the condition that the neurons are driven by the same inputs. We have added now the clarification that details the meaning of this 'restriction': "only those stimulus combinations in which each neuron responded exclusively to one of the two tones; see **Methods** for details." As an aside, including all the stimuli in fact makes little difference to the results because of the trial shuffling corrections.

10. When estimating spike correlations, it is good practice to use signals measured from distinct electrodes to avoid artifacts due to imperfect unit isolation. The authors should explain if the measurements come from the same or different electrodes and make sure unit isolation does not introduce correlation artifacts.

All neuron pairs used for spike correlation analysis were recorded from different electrodes.

11. Frequency is expressed in octaves in the text, but in semitones in the figures. This makes it harder to relate these quantities (for example the frequency of different tones with respect to the response in the STRFs).

Corrected.

12. The figures need to indicate significance levels (p-values) whenever a star is used to indicate significance (see for example, Fig.5B).

Corrected. Added in legends

13. All figures showing STRFs need to have colorbars (see Fig.2), and these colorbars need to have units.

Corrected.

MINOR COMMENTS:
- In Fig.7C, what is zero in the time axis? sounds do not seem to start at zero.

Corrected

- In lines 423-424, it is not clear what the authors mean by "computed from responses to completely segregated responses".

Clarified (as in comment #9 above)

- Line 599 says that several intensities were used, but line 600 does not say which intensity was used for calculating best frequency.

Corrected.

- It would be easier for the reader is panels in Fig.1C where located in Fig.5 where the results are presented.

The placement now is better from the point of view of keeping all paradigm descriptions together for easier comparison. We added a line in the legend of Fig.5, so that readers can easily find the relevant information.

- Line 200: color black dots? The authors probably meant orange stars.

Error is corrected.

- Line 300 says that the PSTH was calculated for 4 conditions, but it is not clear from the figure which plots correspond to each condition.

Clarified both in the text and in the Fig. 7.

- The vertical axis in each inset in Figure 7A needs to be defined.

A black bar was added to indicate the vertical value and unit.

- (Line 133-134) Saying plus/minus and above/below is redundant.

Thank you. Corrected.

- *(Line 145) Typo: "the how" - (Line 270) Close parenthesis. - (Line 295) the words do not correspond to the meaning of PSTH. - (Line 775) Typo: missing words.*

Thank you. All errors corrected.

Reviewer #2:

Specific Comments

The authors interpret the changes they measure in correlations between neurons in terms of the connectivity in the network. This is not unreasonable, but I do not believe they actually provide specific evidence for changes in connection strengths between neurons, since these changes in correlation could be due to changes in the dynamics of the neural activity (e.g., changes in synchrony of presynaptic spikes arriving at synapses of the recorded neuron) even if the synaptic strengths themselves are not changing. They point out that there are reports in the literature of changes in synaptic strength in A1due to short term synaptic plasticity, measured using patch clamp methods, and I agree with them that this is likely to be occurring here, but I think they should make it clear that their results do not prove this; rather, they are consistent with this.

Changed as in Rev.#1 comment #1.

Line 352:"...and hence must have developed rapidly during each trial." I agree that the effect developed rapidly, but I do not think that the average response guarantees that the effect was present in "each" trial.

Our statement is based on the fact that the SYN and ALT trials were *randomly interleaved.* So the effects could not have built up since they would have been cancelled by the random ordering. Instead, when we average the results from each type (SYN or ALT) we get the different adaptive effects in opposite directions. So the simplest conclusion is that they must have occurred on each trial, and that they did not significantly carry over from trial to trial. The reviewer is correct in that we do not know if this pattern happened each and every trial. However, when it happened, it did not persist across trials because if it had, then the SYN and ALT effects would have cancelled each other. This change has been added to the text.

Fig. 3C and D: These scatterplots would be slightly easier for me to view if they were square in shape so the horizontal and vertical scales matched, and if they had thin diagonal lines to indicate equality.

Corrected.

Fig. 4 caption: It took me a minute to see that all plots in the figure are averages across the neural population, so I would add "across all cells" or words to that effect in the figure title, similar to the caption to Fig. 3.

Captions modified as requested.

Fig. 4: It is hard for me to see differences in these plots. It would help if numbers appeared in the corner of each plot (as in Fig. 2) or if color plots of differences were included (as in Fig. 3B).

Numbers added as requested.

Fig. 6B caption, line 934: I believe "proximately" should be "approximately" here.

Corrected

Fig. 6: I do not understand Fig. 6B and C. The caption states that these are differences in the correlations, not correlations of the differences; is that correct? What exactly is being plotted? I would have expected the histograms span a smaller horizontal range than the full range spanned by the original correlation histograms.

They are the distributions of the *differences or changes* in the correlations of each pair between the two conditions (SYN and ALT). After subjecting shuffled trial correlations, the adjusted correlation coefficients were small (around +/- 0.1) so differences of correlation between conditions were approximately of the same magnitude of changes, which makes the histograms span the same horizontal range.

Fig. 7A caption, line 944:The caption mentions red and green vertical lines, but I see no color in Fig. 7A.

The left panel has vertical red lines, and the right panel has the red/green vertical lines indicating the onset of the tones.

Line 121: I think "...it would have resulted in..." should use "might" or "could" here.

Corrected

Line 145: "To assess the how" -> "To assess how"

Corrected

Reviewer #3:

Better defining some of the terms and concepts would strengthen the manuscript. For instance, in the text, rapid changes in neural firing appear to be equated with rapid plasticity. But they are not necessarily the same thing. For instance, changes in neural firing rate could also index attention and/or arousal associated with task demands. It is unclear how one could distinguish between these alternative possibilities. The authors should consider providing an example of a rapid neuroplastic change in the brain or adding a list of prerequisites used to conclude that changes in firing rate index rapid neuroplastic changes rather than rather than attention or arousal.

The reviewer is correct in pointing out that some changes in firing rate may be the result of generalized arousal or attention, and cannot always be assumed to be equated with task-related "plasticity". We also agree that we need better, clearer, operational definitions for terms such as "reward", "arousal", "attention", "plasticity" because there are now quite a wide range of usages and no universally accepted definitions for these important concepts. The essence of our paper is the hypothesis of figure 1, in which we suggest that connectivity changes during task behavior. Over the past fifteen years of our research, we have referred to the rapid changes in spectrotemporal receptive fields (STRFs) in A1 and the associated presumed changes in synaptic connectivity during task performance as “rapid task-related plasticity”. We note that such STRF changes are not always associated with changes in neuronal firing rate (as we have described in earlier publications), even though the animals in our studies are clearly attending to the task. We think it is likely that these changes are indeed the result of attention, and of synaptic modulations due to state changes, but we haven't yet proven this. For us, “rapid task-related plasticity”, measured as a change in receptive fields over a short time-span of seconds or minutes in the context of task performance, that cannot be explained by stimulus-driven adaptation, is simply an adaptive change in the neuron or network that may enhance task performance. We do not yet know the neural mechanisms underlying these observed phenomena. They are likely mediated by rapid synaptic weight changes or perhaps by top-down influences from frontal cortex or other areas, a topic of intense investigation in our lab. But, so as to be consistent with all our previous work since 2003, we understandably wish to maintain this terminology in order not to appear to postulate a different phenomenon in this case. We note that there are many good examples of rapid neuroplastic task-related changes in the brain. We are not sure exactly what the reviewer had in mind in requesting an example, but an example that illustrates the relationship between selective attention and changes in neural firing can be seen in the attentional human ECoG study of Mesgarani and Chang (2012) Nature 485: 233-236 that demonstrated selective cortical representations of the attended speaker in a multi-talker environment.

In the abstract (line 27), there is a distinction made between perceptual streams and sources, "features are segregated into different perceptual stream and sources." The authors should clarify what is the difference

There is no difference between a stream and a source in our conception, so we have removed the word “sources”.

The title of the manuscript leads the reader to expect analyses that focus primarily on functionally connectivity. However, the analyses that was done focuses primarily on the strength of the responses, as

opposed to actual changes in correlations between units. I suggest that the correlation analyses be moved up in the results section because it is the novel element of the study. Agreed. See response to Rev.#1, comment #1

Reorganizing the presentation by having the correlation analysis move to the top (before the STRFs) proved very difficult to follow because prior more visually compelling results like those of Figs. 2,3, proved valuable in clarifying the message of the results before showing them.

Moreover, I think that including more detail regarding the correlation analyses is needed and will improve the paper. For instance, Figure 1 suggests some directionality, but it is unclear (to this reviewer) how direction can be inferred from the correlation analysis.

More details have been added. But, we totally agree with the reviewer on the directionality problem, which could not be fully solved by the current correlation analysis. We have also changed the term “connectivity” to “interaction” in most places (as per reviewer #1 comments).

In the present study, frequency separation between the two tones that composed the sequence is manipulated. Increased frequency may induce changes in loudness perception. This should be discussed.

We acknowledged this fact in **Methods**, and we explained that it could not systematically bias the results in our case because of the accumulation of data from a large number of cells and tests over a large number of frequency combinations.

Also, it is unclear how changing the frequency separation between two tones presented simultaneously can inform us about temporal coherence. For instance, line 227 mentions that temporal coherence of the stimuli gradually decreased as the stimuli became more separated in frequency. How could this be since the timing does not change?

This point was clarified further in the text. What is changing with the frequency separation is not the temporal coherence, but rather its effects on the interactions. The reviewer is absolutely correct that the timing remains the same, but as the frequency separation increases, this coherence becomes less and less effective in altering the interactions.

Line 40, the statement "the physiological mechanisms that underlie this ability remain unknown' is too strong and does not sufficiently acknowledge more than 20 years of research on this topic. Please revise.

Revised. It now says now states: “… yet knowledge of the physiological mechanisms that underlie this ability remains incomplete.”

Line 42, I would replace "perceptual" with "acoustic.".

Corrected

Line 50, perhaps I am misunderstanding, but I was under the impression that sequences of synchronous tones do segregate when the frequency separation between the two tones is large (e.g., one octave separation). This is also what is shown in Elhilali et al. (Neuron, 2009).

In the *Neuron, 2009* paper, the synchronous sequences remained perceived as one stream up to the largest separations used (15 semitones) (Figure 2 and Fig.7B in the *Neuron, 2009* paper).

Line 145, replace "To assess the how" with "To assess how.".

Corrected

Line 354-356, this sentence need to be revised. In humans, the build up of auditory stream segregation usually takes several seconds. To my knowledge, there is little evidence supporting the notion that temporal coherence occurs within 400 ms in human listeners. The references provided by the authors are inaccurate. It is unclear where in Bregman's book the authors found support for a build up of stream segregation in 400 ms. Also, unless I am mistaken, the study from Thompson et al. did not show build up in 400 ms but instead showed build up over several seconds. Please revise, clarify, and/or review carefully that cited work is appropriate (in this section but throughout as well).

We thank the reviewer and provide below more specific references that report segregation estimates that are comparable to the times mentioned in the manuscript. They are

Micheyl, C., Tian, B., Carlyon, R. P., & Rauschecker, J. P. (2005). Perceptual organization of tone sequences in the auditory cortex of awake macaques. *Neuron*, *48*(1), 139-148. Figure 1B shows segregation of two tones almost complete within a second for tones with >=.5 oct separation.

Anstis, S., & Saida, S. (1985). Adaptation to auditory streaming of frequency-modulated tones. *Journal of Experimental Psychology: Human Perception and Performance, 11,* 257–271: Fig. 2, shows build-up with more than 50% "segregated" judgments after about 2 seconds, and that is for frequencies of 800 and 1200, ie only 7 semitones

Micheyl, C., & Oxenham, A. J. (2010). Objective and subjective psychophysical measures of auditory stream integration and segregation. *Journal of the Association for Research in Otolaryngology*, *11*(4), 709-724.: They found streaming after just 2 repetitions of the triple sequence

Pressnitzer et al (2008) Current Biology. Shows both neural and behavioral data for rapid build-up. In Fig. 3, for frequency differences of 6 or 9 semitones, the 50% segregated point is exceeded within about 1 second.

Line 359-361, the sentence is awkward. I would omit "and the rapid adaptive processes that they."

Sentence simplified to: "…and the rapid adaptive processes that potentially play an important role in auditory scene analysis…".

Line 380, the reference is appropriate for MEG but not EEG. That study focused exclusively on MEG data. For an example of a study examining the effect of attention on EEG during stream segregation using ABA pattern, Snyder et al. (2006) would be more relevant and appropriate.

Reference Added.

Line 472, please add the year after Turgeon et al.

Done

Line 530, it is not clear what the difference is between voice and pitch. The ability to focus attention on a particular talker is likely driven primarily by its pitch. Or does voice include other features? Please clarify.

Clarified further to indicate timbre.

Line 733, reference 27, please correct the spelling of the second author. It should be Micheyl. Line 813, please check reference 51, 2016 or in press?

Done

Figure 2, it is unclear what the color coding scale is referring to. Are the panels on the same scale?

Clarified further in text.

Figure 3, I found the figure difficult to understand. Please provide the units for the color bar.

The STRFs here are constructs based on *stimulus*-triggered PSTH's at each line of the spectrogram (see **Methods**). So the units are strictly spikes/sec, except they are normalized, and hence adding the units is misleading. So this is why we leave them out.

We hope you find the revised version and responses to reviewers adequate, and thank you for your consideration of this manuscript.

Sincerely,
Shihab Shamma (on behalf of all authors)
NCOMMS-16-08181-T

<u>Reviewers' comments</u>:

Reviewer #1 (Remarks to the Author):

The authors have addressed most of the concerns raised in the reviews. Below are a few remaining issues that should be addressed:

1. Fig.3C,D: What are the units of the axes?

2. Fig.7A: The caption says: "Red and green vertical lines indicate the onset of each tone in SYN and ALT conditions, respectively", but panel ALT has both green and red lines. Which ones correspond to tone A and which to tone B?

3. Fig.8A: It is intriguing that firing rates during ALT (behaving) are larger than during SYN (behaving), as this seems to contradict results from Fig3. The authors should clarify why the data show this trend. Presumably it has to do with SYN stimuli being two simultaneous tones and ALT only one tone.

4. Line 586 says "All stimuli were presented at 60dBSPL", but line 616 says "All tones were typically played at 70dBSPL". Does the first line refer to all stimuli?

5. Point 8 raised in the first review was address as a reply to reviewers, but it should also be included in the manuscript. Unless all neurons have similar firing rates, averaging rates across neurons is misleading.

Reviewer #2 (Remarks to the Author):

I am satisfied by the authors' responses to my questions and concerns. This study addresses an important question in auditory perception and physiology and I believe these results will be of interest to the readers of Nature Communications.

Reviewer #3 (Remarks to the Author):

The authors have adequately my prior comments. I have some minor suggestions.
line 27, replace principle", with principle,"
line 27, replace fundamental with important
line 320, delete "moderately."

line 158-163, the numbers do not add up, please check.
line 192, and elsewhere, you should have a space between the period and the figure number.
line 200 and elsewhere, make sure the font for the p values is italic.
Line 225-232, what the interaction between near/far and behaviour significant? This is not clear form the description. Please check.

REVIEWERS' COMMENTS:

Reviewer #1 (Remarks to the Author):

The authors have addressed most of the concerns raised in the reviews. Below are a few remaining issues that should be addressed:

1. Fig.3C,D: What are the units of the axes?
Figure legend modified to indicate that axes represent the normalized STRF amplitude as indicated by the color bars.

2. Fig.7A: The caption says: "Red and green vertical lines indicate the onset of each tone in SYN and ALT conditions, respectively", but panel ALT has both green and red lines. Which ones correspond to tone A and which to tone B?
DONE

3. Fig.8A: It is intriguing that firing rates during ALT (behaving) are larger than during SYN (behaving), as this seems to contradict results from Fig3. The authors should clarify why the data show this trend. Presumably it has to do with SYN stimuli being two simultaneous tones and ALT only one tone.

Indeed this is because there are two tone-onsets and responses during a period of the ALT sequence. The sum of these often exceeds that of the responses to the one onset in a SYN period. This of course complicates the interpretation of the response changes. And this is why, in the analysis of spike rates, the comparison between behavior and passive responses are done across the same sequence patterns (SYN or ALT), rather than across patterns.

4. Line 586 says "All stimuli were presented at 60dBSPL", but line 616 says "All tones were typically played at 70dBSPL". Does the first line refer to all stimuli?
It was 60dB. Corrected.

5. Point 8 raised in the first review was address as a reply to reviewers, but it should also be included in the manuscript. Unless all neurons have similar firing rates, averaging rates across neurons is misleading.

We have added now the Wilcoxon signed-rank tests that we alluded to in our response letter earlier to the manuscript.

Reviewer #2 (Remarks to the Author):

I am satisfied by the authors' responses to my questions and concerns. This study addresses an important question in auditory perception and physiology and I believe these results will be of interest to the readers of Nature Communications.

Reviewer #3 (Remarks to the Author):

The authors have adequately my prior comments. I have some minor suggestions.
line 27, replace principle", with principle," **DONE**
line 27, replace fundamental with important **DONE (it is actually line 37)**
line 320, delete "moderately." **DONE (actually line 322)**
line 158-163, the numbers do not add up, please check.

We assume what the reviewer asked for is following content"*The two ferrets participating in these experiments exhibited consistently good performance during the recordings, with the mean percentage of correct trials at 74% and 1% standard error (76%+-1.4% from 36 recordings for one animal, and 71%+-1.7% from 28 recordings for the other). Analysis of correct trials from all 64 recordings found no significant difference in the behavioral performance of the animals following the ALT trials or SYN sequences (repeated measure t-test: p>0.239).*"

We have checked the reported numbers and data and did not find any errors. We are not sure what exactly the reviewer is referring to as "it did not add up". The mean of performance looks right: (0.76*36+0.71*28)/(36+28)=0.74 The standard error for two animals should be smaller than data from either of the single animal as the sample size is bigger.

line 192, and elsewhere, you should have a space between the period and the figure number.
DONE
line 200 and elsewhere, make sure the font for the p values is italic.
DONE

Line 225-232, what the interaction between near/far and behaviour significant? This is not clear form the description. Please check.

We have now added a three-way ANOVA to clarify that there is a significant interaction among NEAR/FAR, SYN/ALT and PASSIVE/BEHAVIOR. Please see p223-228.
